# Combining detrended cross-correlation analysis with Riemannian geometry-based classification for improved brain-computer interface performance

**DOI:** 10.3389/fnins.2024.1271831

**Published:** 2024-03-14

**Authors:** Frigyes Samuel Racz, Satyam Kumar, Zalan Kaposzta, Hussein Alawieh, Deland Hu Liu, Ruofan Liu, Akos Czoch, Peter Mukli, José del R. Millán

**Affiliations:** ^1^Department of Neurology, Dell Medical School, The University of Texas at Austin, Austin, TX, United States; ^2^Department of Physiology, Faculty of Medicine, Semmelweis University, Budapest, Hungary; ^3^Mulva Clinic for the Neurosciences, Dell Medical School, The University of Texas at Austin, Austin, TX, United States; ^4^Chandra Family Department of Electrical and Computer Engineering, Cockrell School of Engineering, The University of Texas at Austin, Austin, TX, United States; ^5^Oklahoma Center for Geroscience and Healthy Brain Aging, University of Oklahoma Health Sciences Center, Oklahoma City, OK, United States; ^6^Vascular Cognitive Impairment and Neurodegeneration Program, Department of Neurosurgery, University of Oklahoma Health Sciences Center, Oklahoma City, OK, United States; ^7^International Training Program in Geroscience, Doctoral School of Basic and Translational Medicine/Department of Public Health, Semmelweis University, Budapest, Hungary; ^8^Department of Biomedical Engineering, Cockrell School of Engineering, The University of Texas at Austin, Austin, TX, United States

**Keywords:** brain-computer interface, detrended cross-correlation analysis, Reimannian geometry, motor imagery, detrended fluctuation analysis, fractal connectivity, online

## Abstract

Riemannian geometry-based classification (RGBC) gained popularity in the field of brain-computer interfaces (BCIs) lately, due to its ability to deal with non-stationarities arising in electroencephalography (EEG) data. Domain adaptation, however, is most often performed on sample covariance matrices (SCMs) obtained from EEG data, and thus might not fully account for components affecting covariance estimation itself, such as regional trends. Detrended cross-correlation analysis (DCCA) can be utilized to estimate the covariance structure of such signals, yet it is computationally expensive in its original form. A recently proposed online implementation of DCCA, however, allows for its fast computation and thus makes it possible to employ DCCA in real-time applications. In this study we propose to replace the SCM with the DCCA matrix as input to RGBC and assess its effect on offline and online BCI performance. First we evaluated the proposed decoding pipeline offline on previously recorded EEG data from 18 individuals performing left and right hand motor imagery (MI), and benchmarked it against vanilla RGBC and popular MI-detection approaches. Subsequently, we recruited eight participants (with previous BCI experience) who operated an MI-based BCI (MI-BCI) online using the DCCA-enhanced Riemannian decoder. Finally, we tested the proposed method on a public, multi-class MI-BCI dataset. During offline evaluations the DCCA-based decoder consistently and significantly outperformed the other approaches. Online evaluation confirmed that the DCCA matrix could be computed in real-time even for 22-channel EEG, as well as subjects could control the MI-BCI with high command delivery (normalized Cohen's κ: 0.7409 ± 0.1515) and sample-wise MI detection (normalized Cohen's κ: 0.5200 ± 0.1610). *Post-hoc* analysis indicated characteristic connectivity patterns under both MI conditions, with stronger connectivity in the hemisphere contralateral to the MI task. Additionally, fractal scaling exponent of neural activity was found increased in the contralateral compared to the ipsilateral motor cortices (C4 and C3 for left and right MI, respectively) in both classes. Combining DCCA with Riemannian geometry-based decoding yields a robust and effective decoder, that not only improves upon the SCM-based approach but can also provide relevant information on the neurophysiological processes behind MI.

## 1 Introduction

Brain-computer interfaces (BCIs) establish a link between the central nervous system and some external device, thus allowing the user to deliver commands via brain patterns exclusively and without any involvement of the peripheric nervous or skeletomuscular systems (Wolpaw et al., 2020). By far the most popular modality for non-invasive brain computer interfacing is electroencephalography (EEG) due to its high temporal resolution, flexibility and relatively low cost. With recent advancements in both the software and hardware domains, EEG-based BCIs already paved their ways to a wide variety of applications, including but not limited to spelling devices (Yin et al., 2014; Chavarriaga et al., 2016), robotics control (Gordleeva et al., 2017; Beraldo et al., 2022; Mitra et al., 2023) or neurorehabilitation (Biasiucci et al., 2018). Nevertheless, although EEG appears as a suitable choice of imaging technique for a non-invasive BCI, it also has its set of shortcomings. EEG has limited spatial resolution and imprecise anatomical localization, low signal-to-noise ratio due to electric field attenuation, and is also susceptible for a range of artifacts, eventually resulting in a fairly unstable signal. This instability is one of the main reasons why most EEG-based BCIs have poor generalizability both over longer time periods and among different individuals, and modest overall performance, especially in contrast with their invasive counterparts (Steyrl et al., 2016).

Consequently, immense efforts were made aiming at more reliable non-invasive BCI performance, ranging from the development of novel artefact-removal techniques (Kim and Kim, 2018) to the adaptation and production of sophisticated decoding algorithms (Xu et al., 2021). Among the latter, Riemannian geometry-based classification approaches are gaining much popularity lately, mainly due to their computational efficiency and favorable properties in dealing with non-stationarities widely present in EEG data (Congedo et al., 2017; Zanini et al., 2017). Briefly, Riemannian geometry-based classifiers operate with symmetric positive definite (SPD) matrices as input and allow for domain adaptation, reducing non-stationarities arising from covariance shifts between different recording sessions (Kumar et al., 2019, 2024). Sample covariance matrices (SCMs) are SPD and can be easily obtained from empirical EEG data, hence allowing for Riemannian geometry-based classification of neural signals (Barachant et al., 2010b). Nevertheless, the performance of Riemannian geometry-based approaches yet fundamentally depend on the covariance estimate itself, which can be greatly affected by local non-stationarities present in the data.

Podobnik and Stanley (2008) proposed detrended cross-correlation analysis (DCCA) to assess the covariance between a pair of non-stationary signals and the plausible power-law (or *fractal*) nature of their long-range coupling. DCCA employs a local detrending step that ideally renders smaller segments of the given data set stationary, thus promoting the estimation of the covariance structure absent from the biasing effects of regional non-stationarities. Furthermore, in case of multivariate signals, performing DCCA in a pairwise manner yields SPD matrices (as demonstrated below). Recent studies also indicate that the concept of DCCA could be utilized effectively to capture relevant functional connectivity patterns in the human brain (Chen et al., 2018; Ide and Chiang-shan, 2018; Kaposzta et al., 2023). Therefore, these notions render DCCA as a potentially attractive candidate in providing input for Riemannian geometry-based classification schemes of neural data. In its original formulation DCCA is computationally expensive, which contradicts its applicability in online BCI applications where real-time performance is essential. Recently, however, Kaposzta et al. (2022) proposed an online formula for DCCA (rtDCCA) allowing for real-time computation of the DCCA matrix from multivariate data and thus resolving this limitation.

Accordingly, in this paper we propose a novel BCI decoding pipeline combining rtDCCA with Riemannian geometry-based classification, where the SCM is replaced with a DCCA matrix as classifier input. To assess the utility of this approach, we first evaluate it offline and pseudo-online on two—one independent and one in-house—previously recorded and validated datasets employing motor imagery (MI) paradigms and benchmark it against a vanilla Riemannian geometry-based pipeline and a standard decoder utilizing common spatial patterns (CSP) and linear discriminant analysis (LDA) for detecting MI (Blankertz et al., 2007). Then, we also test our pipeline in a true online setting employing the same MI paradigm, demonstrating for the first time that the rtDCCA formula can indeed be utilized in a real-time BCI application. Our offline results suggest that replacing the sample covariance matrix with DCCA improves decoding accuracy, also surpassing the acknowledged CSP-based method. Furthermore, we show that the rtDCCA formula can be used in real time to create a robust BCI decoder that operates online at high performance even when trained on a relatively small dataset. Finally, we conduct *post-hoc* analyses to assess how fractal connectivity and regional neural dynamics are affected by left and right MI.

## 2 Materials and methods

### 2.1 Participants and experimental procedures

As mentioned before, we verified our proposed approach of combining DCCA with Riemannian geometry-based classification on three independent data sets. First, we evaluated the detection pipeline offline (and pseudo-online) on previously collected data in order to assess its potential effects on performance in an MI task. Then, we tested if rtDCCA (Kaposzta et al., 2022) can indeed be utilized online in a BCI application. Finally, we further validated our proposed method on an independent, publicly available dataset (Schalk et al., 2004). Below we provide specifics and details of these datasets. All in-house recordings took place at the Engineering Education and Research building and at the Health Discovery Building at The University of Texas at Austin, involving young, healthy volunteers. The studies were conducted in line with the guidelines of the Declaration of Helsinki, and were reviewed and approved by the institutional ethics committee (approval number: 2020-03-0073). All participants provided written informed consent prior to the recordings.

The in-house offline MI dataset consisted of EEG data collected from 18 young, healthy volunteers (age: 23.22 ± 3.59 years, seven female) performing a motor imagery (MI) task involving imagined movements with the left or right hand. None of the participants had experience with operating a BCI before. EEG was recorded at 512 Hz with an ANT Neuro EEGO device (ANT Neuro, Netherlands) from 32 standard scalp locations according to the international 10–10 system, however only data from 22 channels positioned around sensorimotor areas (F7, F3, Fz, F4, F8, FC5, FC1, FC2, FC6, C3, Cz, C4, CP5, CP1, CP2, CP6, P7, P3, Pz, P4, P8, and POz) were used for further analysis. Synchronized electrooculography (EOG) signals were collected via a bipolar box connected to the amplifier, from three channels according to the triangular montage described in Schlögl et al. (2007). Ground and reference electrodes were positioned at AFz and CPz, respectively, and electrode impedances were kept under 25 kΩ. EEG data for the online demonstration was collected using the exact same setup as for the offline dataset. For these recordings we recruited eight young, healthy individuals (age: 26.38 ± 6.21 years, one female) with previous experience in MI. None of the participants in either group had been ever diagnosed with any medical condition affecting the central nervous system, or were under any medication that might influence their BCI performance.

**Figure 1:**
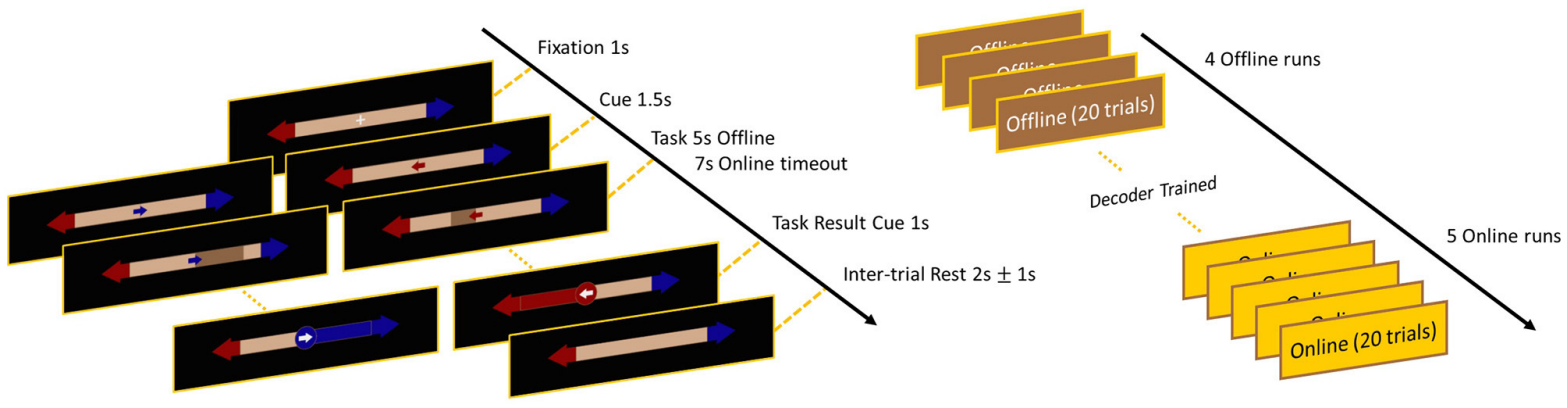
Timeline and visual illustration of the employed bar feedback task. The left panel illustrates the visual interface and feedback provided for the subjects, while the right panel shows the experimental design of the offline and online sessions. Note that in the offline dataset the same visual interface was used, only subjects performed four offline runs on the first day and four online runs on the second day.

Participants performed the benchmark bar feedback BCI task (Leeb et al., 2013), during which the user has to imagine movements with their left or right hands and feedback is provided by a horizontal bar growing in the direction of the motor intent. The task and the experimental design is illustrated on Figure 1. A single trial (both offline and online) started with a fixation cross at the center of the screen (presented for 1 s), that was followed by the cue corresponding to the MI-class (left/right) of the upcoming trial (presented for 1.5 s). In the offline setting, this was followed by a visual guidance cue, where a one dimensional horizontal bar was filling up toward the given MI direction in 5 s (Figure 1, left). Participants were instructed to perform an imaginary movement with their corresponding arm during this period. The nature of movement was not explicitly specified as in subjects were free to decide what type of movements they imagined with their left/right arms, however it was requested to remain consistent throughout the recording. During all recordings, an investigator was observing the participants continuously to ensure that they indeed did not execute any motions during active trial periods. In the online setting, the horizontal bar was utilized to provide real-time visual feedback on the users' neural patterns. Bar size was updated every 62.5 ms showing the accumulated probability of EEG activity belonging to a given MI class, until the user reached a pre-defined threshold value (of either the correct or the erroneous class), or the trial timed out after 7 s (see Section 2.4.2. below for more details).

In the offline dataset, each participant completed a calibration session and an online task performance session on two consecutive days. Note that this design introduced between-session non-stationarities (e.g., slightly different electrode placements and impedances) that increase the challenge of the decoding task, as elaborated on later. One recording session consisted of four runs of MI task performance, with one run comprising 20 trials—10-10 for left and right directions—in a randomized order. This dataset was also analyzed and verified in previous studies (Liu et al., 2023; Kumar et al., 2024). In the online demonstration, subjects performed four offline runs to collect calibration data for training the decoder, then performed five runs online on the same day (Figure 1, right), hence to reduce the effects of between-session non-stationarities. The entire recording session lasted no more than 90 minutes.

Finally, we utilized the EEG Motor Movement/Imagery Dataset version 1.0.0 (Schalk et al., 2004) made available via Physionet (Goldberger et al., 2000) at https://physionet.org/content/eegmmidb/1.0.0/ to assess the performance of our method on an independent sample. Here we only provide a brief description of the dataset, while for further details the reader is referred to the data descriptor. The dataset consisted of EEG recordings from 109 young, healthy volunteers; however data of only 103 participants were analyzed after excluding incomplete entries (Shuqfa et al., 2023). EEG was collected from 64 standard 10–10 locations at a sampling rate of 160 Hz. To remain consistent in our analytical approaches, we analyzed data from the same exact 22 channels as described previously. Participants performed two separate MI paradigms: (*i*) left vs. right hand MI where they had to imagine squeezing their left or right hand, respectively, and (*ii*) both hands vs. both feet MI where they were instructed imagining squeezeing with both their hands or feet, respectively. For each participant, all data was collected in a single recording session, where they performed three runs for both MI paradigms. Each run consisted of 15 trials—8 and 7 for the two conditions in a counterbalanced manner—and each trial lasted for 4.1 s. In the first paradigm, left/right MI was indicated by arrows on the corresponding sides of a computer screen, while in the latter both hands and both feet MI was instructed via arrows on the top and on the bottom of the screen, respectively. Note that this dataset only consisted of offline recordings, i.e., online feedback on neural activity was not provided to the users at any time.

### 2.2 Riemannian geometry-based classification

Riemannian geometry-based classification became increasingly popular lately in BCI applications due to its simple nature and robustness against non-stationarities (Yger et al., 2016; Congedo et al., 2017; Kumar et al., 2019). Here we briefly summarize the essence of Riemannian geometry-based decoding in a generic two-class scenario, while for more details on its mathematical background we refer the reader to the cited works below.

Let us denote the set of symmetric matrices of size *n* × *n* as $S_{n}$ and that of size *n* × *n* symmetric positive definite (SPD) matrices as $P_{n}$. Given that covariance matrices are SPD, any sample covariance matrix *C* estimated from *n*-channel EEG data will therefore be $C\in S_{n}$ and $C\in P_{n}$ for ∀*n*. A *Riemannian manifold* is defined as a smooth manifold equipped at any point with a finite dimensional Euclidean tangent space that is homogeneous to $S_{n}$. Due to their SPD nature, covariance matrices lie on the Riemannian manifold (Moakher, 2005; Yger et al., 2016). On the manifold, the shortest path (also called a geodesic) between two SPD matrices $P_{1},P_{2}\in P_{n}$ can be defined as Barachant et al. (2010b):


(1) $$\begin{array}{l} \gamma \left(P_{1},P_{2},t\right)=P_{1}^{\frac{1}{2}}{\left(P_{1}^{-\frac{1}{2}}P_{2}P_{1}^{-\frac{1}{2}}\right)}^{t}P_{1}^{\frac{1}{2}},\;t\in \left[0,1\right]. \end{array}$$

that can be then utilized to estimate their distance δ(*P*_1_, *P*_2_) according to Moakher (2005):


(2) $$\begin{array}{l} \delta \left(P_{1},P_{2}\right)=\int_{0}^{1}\gamma \left(P_{1},P_{2},t\right)dt=\|\log\left(P_{1}^{-\frac{1}{2}}P_{2}\right){\|}_{F} \end{array}$$

where log(·) refers to matrix logarithm and F is the Frobenius norm of the resulting matrix. The distance metric defined in Equation 2 and derived from the geodesic (Equation 1) is invariant under affine transformations on the Riemannian manifold (Moakher, 2005), and thus is often referred to as the Affine Invariant Riemannian Metric (AIRM). Furthermore, given a set of SPD matrices on the Riemannian manifold, their Karcher mean $\bar{P}$ (center of mass, also refrerred to as Riemannian mean interchangeably) can be obtained as the SPD matrix that minimizes the squared AIRM distance between all SPD matrices in the set (Kumar et al., 2019):


(3) $$\begin{array}{l} \bar{P}=\underset{P\in P_{n}}{\arg\min}\overset{N}{\underset{i=1}{\sum }}\delta^{2}\left(P_{i},P\right). \end{array}$$

The concepts of Riemannian mean and distances could be utilized to build a minimal distance to the mean (MDM) classifier as proposed by Barachant et al. (2010b). Let us consider an EEG dataset consisting of samples (i.e., EEG epochs) from two classes (e.g., left vs. right MI). First, samples are characterized by their covariance structure, such as via their sample covariance matrices. Then, class prototypes ${\bar{C}}_{1}$ and ${\bar{C}}_{2}$ are computed using Equation 3 for the two classes separately. Finally, for every new incoming sample (after transformed into SPD covariance matrix) the AIRM distance to both class prototypes is computed, and the class corresponding to the prototype with smaller distance is predicted. Note that these outlined concepts can be generalized trivially for more than two classes, too.

It is commonly accepted that EEG data is non-stationary between different subjects, but even between different recording sessions of the same subject (Raza et al., 2016). Furthermore, EEG is often considered as non-stationary over longer time scales (Kaplan et al., 2005). These non-stationarities will result in greatly differing distributions of the estimated sample covariance matrices across different sessions/days even within a single subject, which in turn will lead to unstable decoding of neural correlates on the grand scale. However, as proposed by Zanini et al. (2017), due to the affine invariant property of the Riemannian distance metric (Equation 2) covariance matrices from multiple sessions can be aligned to have a similar distribution. At the same time, this step preserves the within session structure, and thus provides robustness against non-stationarities introduced by covariance shifts. These properties made the Riemannian geometry-based approach very attractive in BCI applications lately (Yger et al., 2016; Congedo et al., 2017). Nevertheless, domain adaptation can only align sample covariance matrices once they are estimated from data, and therefore it does not necessarily compensate for all effects that might affect covariance estimation itself. In this paper, we propose DCCA to replace sample covariance estimation to ameliorate this issue and provide further robustness to Riemannian geometry-based classification via removing local non-stationarities from the empirical EEG signal, as elaborated on in the followings.

### 2.3 Detrended cross-correlation analysis

DCCA was first introduced by Podobnik and Stanley (2008) as a tool to estimate the covariance between two non-stationary signals and to assess the potential power-law nature of their coupling. It is a bivariate extension of Detrended Fluctuation Analisys (DFA), a popular tool to characterize fractal temporal scaling in univariate signals (Peng et al., 1995).

Given two long-range coupled time series *x*(*t*) and *y*(*t*) of length *N*, first their integrated versions *X*(*t*) and *Y*(*t*) are computed according to


(4) $$\begin{array}{l} \begin{array}{c} X\left(t\right)=\overset{t}{\underset{i=1}{\sum }}x\left(i\right)\\ Y\left(t\right)=\overset{t}{\underset{i=1}{\sum }}y\left(i\right). \end{array} \end{array}$$

Then, at given scale *s* the integrated time series are divided into *K* = *N* − *s* + 1 overlapping windows containing *s* values each, with at *t* = *k* starting at *k* and ending at *k* + *s* − 1. For both time series the local trends ${\tilde{X}}_{k}$ and Ỹ_*k*_ are estimated independently in each window *k* via ordinary least squares (OLS) regression, and subtracted from the data. Covariance of the residuals in each window are then estimated according to


(5) $$\begin{array}{l} f_{DCCA}^{2}\left(s,k\right)=\frac{1}{s-1}\overset{k+s-1}{\underset{i=k}{\sum }}\left(X\left(i\right)-{\tilde{X}}_{k}\left(i-k+1\right)\right)\left(Y\left(i\right)-{Ỹ}_{k}\left(i-k+1\right)\right) \end{array}$$

and finally the estimate of detrended covariance at scale *s* is obtained via averaging over all windows (Podobnik and Stanley, 2008; Podobnik et al., 2011):


(6) $$\begin{array}{l} F_{DCCA}^{2}\left(s\right)=\frac{1}{K-1}\overset{N-s1}{\underset{k=1}{\sum }}f_{DCCA}^{2}\left(s,k\right). \end{array}$$

Even though the procedure outlined in Equations 4–6 describes a sliding window approach with a step size of one data point, it is more common to obtain $F_{DCCA}^{2}\left(s\right)$ by dividing the time series into *K*_*NO*_ = *floor*(*N*/*s*) non-overlapping windows for computational efficiency. Also note that by making *x*(*t*) = *y*(*t*) one arrives at the well-known formula for DFA.

Extending the univariate approach proposed for DFA by Hartmann et al. (2013) it has been shown that by rearranging the terms in the closed form solution for OLS regression and by computing covariance in a one-pass manner, $F_{DCCA}^{2}\left(s\right)$ can be obtained in real-time (referred to as rtDCCA) for a pair of incoming signals (Kaposzta et al., 2022). Furthermore, in case of multivariate signals the rtDCCA formula can be expressed as a set of matrix operations, rendering the computation of a DCCA matrix very efficient. Therefore, the online algorithm proposed by Kaposzta et al. (2022) is ideal to obtain the detrended covariance structure of incoming EEG signals in real time. Implementations of rtDCCA in Matlab and Python are available at https://github.com/samuelracz/rsDCCA.

Since DCCA in essence computes a covariance matrix (see Equation 5), the resulting matrices are by definition SPD (also considering that the sum/average of two SPD matrices is also SPD). Therefore, DCCA could be easily combined with the Riemannian geometry-based classification approach. Furthermore, since DCCA was proposed to capture the covariance structure of coupled processes with local (or global) non-stationarities (Podobnik and Stanley, 2008)—a property often characteristic for EEG signals (Kaplan et al., 2005)—, this would make it an ideal fit for extracting features from neural data in BCI applications.

Two notions must be stressed, however. First, in the proposed approach our main goal at this point is to exploit the “denoising” effect of local detrending by eliminating non-stationarities, and not to characterize the plausible fractal nature of long-range coupling between the neural activity of various brain regions [such as in e.g., Chen et al. (2018)]. Therefore, in our analyses we omit the first integration step (Equation 4) and instead compute the covariance of residuals from native EEG data to substantiate the denoising effect. Second, even though local detrending can eliminate/reduce non-stationarities, depending on the scale *s* it can act similar to a high-pass filter, and thus can also remove valuable information. Therefore, it is impediment to explore the best choice of *s* that indeed improves and not hampers decoding performance.

### 2.4 Data pre-processing, analysis strategy and decoding

All data analysis steps were carried out using Matlab 2021b (The Mathworks, Nattick, MA, USA) and Python 3.7.3. The BCI user interface was developed in Python using the PyGame library.

#### 2.4.1 Offline MI dataset

Raw EEG data was first band-pass filtered using a 3rd order, zero-phase Butterworth filter with cutoff frequencies 8 and 30 Hz, as sensorimotor rhythms have been established as being modulated during MI performance (Yger et al., 2016; Rimbert and Lotte, 2022; Shuqfa et al., 2023). Active MI periods (6.5-s segments from the offline, while segments of varying length from the online recording session) were isolated, and then further segmented into 1-s long epochs, simulating a sliding window analysis with a step size of 62.5 ms (i.e., 93.75% overlap among consecutive windows in a trial).

We evaluated decoding performance on the MI dataset in three separate pipelines, where decoders were trained on data from the first session, and performance was evaluated on data from the second session:

- Riemannian-MDM: We utilized the standard Riemannian-MDM classification pipeline as outlined in e.g., Kumar et al. (2019, 2024). In that, trace-normalized covariance matrices for each 1 s epoch were estimated as input features using the shrinkage method proposed by Ledoit and Wolf (2004). Decoding was then performed by an MDM classifier, where class prototypes (Riemannian means of matrices belonging to each class) were obtained via a gradient descent-based iterative process (Barachant et al., 2010b).
- DCCA-Riemannian-MDM: In this case features were estimated using DCCA instead of sample covariance. In order to assess the plausible effect of scale (as outlined previously), we completed this analysis at five different scales with *s* = 32, 64, 128, 256, and 512 data points (corresponding to 62.5 ms, 125 ms, 250 ms, 0. 5 s, and 1 s window sizes for local detrending). Even though rtDCCA allows for rapid multiscale estimation (Kaposzta et al., 2022) yielding tensors of size *N*_*ch*_ × *N*_*ch*_ × *N*_*s*_ (with *N*_*ch*_ and *N*_*s*_ denoting the number of channels and number of detrending scales, respectively), for the sake of simplicity in this study we only utilized single-scale DCCA matrices for epoch classification. Decoding was performed using an MDM classifier similarly to the Riemannian-MDM pipeline.
- Cov-CSP-LDA: As a benchmark classification method, we evaluated decoding performance using common spatial patterns (CSP) (Blankertz et al., 2007). Specifically, first the sample covariance matrix of each epoch was estimated as in the Riemannian-MDM pipeline. Then, spatial filters were estimated from covariance matrices using the method proposed by Barachant et al. (2010a). Finally, the three most discriminative filters were used as features for classification using linear discriminant analysis (LDA).

Furthermore, to assess how these decoding approaches would perform not only in an offline but also in an online setting, they were executed under three evaluation schemes:

- Offline: The obtained features (sample covariance matrices, DCCA matrices and sample covariance-derived CSPs) were used unaltered for decoder training and testing.
- Rebiasing: In this scenario, we utilized standard rebiasing (Zanini et al., 2017) for the initial covariance or DCCA matrices [see the *Rebias MDM* pipeline in Kumar et al. (2019) for details]. Specifically, in each pipeline a global reference matrix *R* was obtained as the Karcher mean of all matrices *C*_*i*_ regardless of class or session (He and Wu, 2019) that was then used to re-center *C*_*i*_ in the entire dataset according to
  (7) $$\begin{array}{l} C_{i}^{rebias}=R^{-\frac{1}{2}}C_{i}R^{-\frac{1}{2}}. \end{array}$$
  Subsequently, training and testing (including CSP estimation) were carried out using the rebiased matrices.
- Adaptive rebiasing: This setup was conducted to emulate an online setting. In that, reference matrices *R*^*train*^ were obtained using only matrices from the training set, and decoders were trained using data rebiased according to Equation 7. However, the now unknown reference matrices *R*^*test*^ for the test sets were estimated in an adaptive manner, maintaining the temporal causality of the incoming epochs (Kumar et al., 2019, 2024). Precisely, *R*^*test*^ was updated for each incoming test example $C_{i}^{test}$ according to
  (8) $$\begin{array}{l} R_{i}^{test}=\{\begin{array}{l} R^{train}\;i=1\\ C_{i-1}^{test}\;i=2\\ \gamma (R_{i-1}^{test},C_{i}^{test},\frac{1}{i-1})\;i>=2 \end{array} \end{array}$$

Evaluation (including CSP estimation) was conducted on adaptively rebiased test data by substituting $R_{i}^{test}$ in Equation 7.

#### 2.4.2 Online MI demonstration

Raw EEG and EOG data was received every 62.5 ms (1/16 s) in packets of 32 data points and band-pass filtered online using a 2^*nd*^ order causal Butterworth filter with cutoff frequencies 8–30 Hz and 1–10 Hz for EEG and EOG, respectively. Neural activity was decoded online at the same rate always using the latest 1 s of incoming EEG. Incoming trials were rejected from decoding if blinks or large, sudden eye movements were detected in the EOG (Perdikis et al., 2018), i.e., if absolute EOG amplitude surpassed a pre-defined threshold value (320 μV). Note, that this pipeline implies that all pre-processing steps and the computation of 22×22 DCCA matrices could be completed under 62.5 ms. All recordings were carried out using a personal computer with Intel Core i7-8665U CPU (1.90 Ghz × 8) and 16 Gb RAM, running on Ubuntu 18.04 operating system.

EEG classification was carried out using the following decoder architecture. The offline data was used to train the subject-specific MDM decoder for each participant using rebiased DCCA matrix input features as described for the offline dataset. In the online setting, DCCA was computed using the rtDCCA formula from each incoming, pre-processed EEG epoch of 1 s using *s* = 128 data points for local detrending (see results). The obtained matrices were then adaptively re-centered online according to Equation 8 (as in Kumar et al. (2024)) and predictions were made by the MDM decoder. The exponentially smoothed prediction probability *Prob*_*i*_ was used as the online visual feedback (Leeb et al., 2013) at time instance *i* according to


(9) $$\begin{array}{l} Prob_{i}=\left(1-\alpha \right)Prob_{i-1}+\alpha Prob_{i} \end{array}$$

where the smoothing factor was set as α = 0.05. Initial probabilities were reset to a uniform distribution (*Prob*_0_ = 0.5) before the start of each trial. A trial terminated and the appropriate feedback is shown for 2 s if (*i*) the accumulated prediction evidence surpassed a pre-defined threshold in the right direction (correct command), (*ii*) it surpassed the threshold in the opposite direction (erroneous command), or (*iii*) the user was unable to reach any of the thresholds in 7 s (timeout). Thresholds were set independently for each run and separately for both directions, adapting to the user's performance in order to minimize erroneous command delivery while at the same time provide enough challenge so that maintain user involvement (Leeb et al., 2013; Biasiucci et al., 2018). Each trial was followed by an inter-trial rest period randomized in the range of 2 ± 1 s.

#### 2.4.3 Independent MI dataset

Pre-processing of the independent dataset was carried out following the same exact steps as described previously for our in-house offline data. We evaluated these recordings in three settings: (*i*) two-class left vs. right hand MI, (*ii*) two-class both hands vs both feet MI, and (*iii*) four-class MI including all conditions. To remain consistent with our prior analyses, we segmented the pre-processed recordings the following way. First, active MI periods were isolated, then epoched into 1-s long segments with a step size of 62.5 ms (10 data points at 160 Hz). Since these recordings were performed in a single session, we utilized a leave-one-run out cross-validation (LORO-CV) scheme for evaluation in case of every participant individually, where in each iteration all examples from one run was used as the holdout set for testing, while models were trained in the examples from the remaining two runs. In the four-class MI setting, one left/right hand MI and one both hands and both feet MI runs were used collectively as the holdout set. Finally, performance measures (see below) were averaged over the LORO-CV runs to obtain subject-wise outcomes. In all three settings, data was analyzed employing the same three decoding approaches (Riemannian-MDM, DCCA-Riemannian-MDM and Cov-CSP-LDA) and evaluation schemes (Offline, Offline Rebias and Adaptive Rebias) as described previously, with the adjustment that when using the DCCA-Riemannian-MDM decoder, DCCA was computed at detrending scales *s* = 10, 20, 40, 80, and 160 corresponding to the same window sizes as utilized for 512 Hz data.

### 2.5 Performance evaluation

We utilized the following metrics to characterize decoder performances:

- Sample-wise accuracy, Cohen's Kappa and Bar dynamics: Accuracy is computed for each 1 s epoch individually as Equation 10:
  (10) $$\begin{array}{l} Acc=\frac{TP+TN}{TP+TN+FP+FN}, \end{array}$$
  with *TP*, *TN*, *FP* and *FN* denoting the number of true positives, true negatives, false positives and false negatives, respectively (Fawcett, 2006). Note that predictions are obtained using raw outputs from the decoder, and not accumulated evidence. Chance levels for accuracy were computed assuming a binomial distribution of prediction errors as proposed by Combrisson and Jerbi (2015) at a confidence level of *p* = 0.001. Cohen's Kappa (κ) also serves as a measure of classification accuracy (Cohen, 1960), however, it provides a better assessment of performance compared to chance level in case of unbalanced samples, such as in case of online decoding, where the number of epochs for each MI class depends on how fast the user reaches the threshold. Cohen's Kappa ranges from −1 to 1 with κ = 1 implying perfect classification, while κ = 0 indicating chance level performance. We characterized performance according to the ranges suggested by Landis and Koch (1977), with ranges κ ∈ [−1;0], (0 − 0.2], (0.2 − 0.4], (0.4 − 0.6], (0.6 − 0.8] and (0.8 − 1] indicating random, poor, fair, moderate, substantial and perfect performance, respectively. Finally, bar dynamics refer to the proportion of epochs (given as percentage) when the user was able to drive and keep the bar on the correct direction in a given trial. In other terms, bar dynamics is equivalent to sample-wise accuracy estimated from accumulated evidence instead of raw decoder output.
- Command delivery: This measure characterizes the discrete, command-level performance of the user based on the accumulated evidence defined in Equation 9. Command delivery was characterized by the Kappa value normalized to the number of timeouts (κ_*norm*_); in which κ is computed from the confusion matrix constructed from correct and incorrect commands (excluding timeouts) and then normalized according to Equation 11:
  (11) $$\begin{array}{l} \kappa_{norm}=\kappa *\left(1-\frac{n_{timeouts}}{n_{total}}\right) \end{array}$$
  where *n*_*timeouts*_ and *n*_*total*_ denote the number of timeout and completed trials, respectively. We also computed accuracy of command delivery with (*Acc*_*approx*_) and without (*Acc*_*comp*_) including trials resulting in timeout. In the former case, the command was determined by the direction of the bar at the instance of the timeout.

Performance in the offline datasets was characterized only with sample-wise accuracy and Cohen's κ, while online BCI control was assessed additionally with sample-wise bar dynamics and command delivery measures. Note that neither sample-wise measures, nor normalized command delivery metrics are affected by the threshold value set throughout the recordings. When comparing the performance of the various pipelines in the offline evaluation, the main effect (Riemannian-MDM vs. DCCA-Riemannian-MDM vs. Cov-CSP-LDA) was assessed via Friedman's test, as in most cases data did not satisfy the condition of normality as confirmed by Lilliefors test. If a main effect was identified, *post-hoc* pairwise comparisons were carried out using Wilcoxon signed rank tests, and outcomes were adjusted for multiple comparisons using the False Discovery Rate (FDR) method of Benjamini and Hochberg (1995).

### 2.6 Extracting neural patterns

The main goal of BCI research is to provide working applications for individuals in need; however, unveiling the neurophysiological underpinnings of the utilized paradigms are just as important. Therefore, we re-analyzed the data collected in the online MI setting to assess how the brain adapts when performing left and right imagery of movements. The pre-processing strategy followed that described for offline data analysis, with the addition that only successful trials (i.e., trials resulting in correct left or right command delivery) were considered to enhance signal-to-noise ratio (Shin et al., 2018). For each subject, the measures obtained from each epoch were finally collapsed via averaging to provide the characteristic (subject-specific) grand average for both MI classes, that were then subjected to group-level statistics. Note that during the statistical evaluation we did not adjust for multiple comparisons, and therefore these results should be considered as exploratory.

#### 2.6.1 Fractal connectivity patterns during MI task performance

In general, DCCA, is utilized for two purposes: (*i*) to assess the covariance of non-stationary signals and (*ii*) to characterize the plausible power-law nature of their long-range dependence (Podobnik and Stanley, 2008). In this regard, a DCCA matrix can be understood as estimating the functional connectivity (FC) of the brain to some extent, i.e., capturing the statistical relationship between activity of two cortical regions (Bullmore and Sporns, 2009). Therefore, we compared the DCCA structure of left-MI to right-MI. Keeping in mind, however, that the absolute value of covariance in itself (and thus DCCA) does not necessarily reflect the strength of functional cooperation given that the measure is unbounded and primarily influenced by the variance of the individual signals. To resolve this issue, DCCA was further developed into the detrended cross-correlation coefficient (DCCC) by Zebende (2011). It can be shown that this measure is indeed bounded between −1 and 1 as a regular correlation coefficient (Podobnik et al., 2011), and its value is thus proportional to the strength of coupling. We reconstructed the DCCC networks for left and right MI at detrending scale *s* = 128 (as based on classification results, see below) and compared the obtained values in a connection-to-connection manner. EEG data was pre-processed the same as described previously for offline analysis. Connections in the two MI conditions were contrasted individually using paired *t*-test or Wilcoxon signed rank test, depending on the normality of the data as assessed via Lilliefors test. We sorted those connections that were identified as significantly different into three categories: (*i*) connections that are positively correlated (DCCC ∈ [0;1]) in both left and right MI, (*ii*) connections that are negatively correlated (DCCC ∈ [−1;0]) in both conditions, and (*iii*) connections that are positive/negative in one condition, but the opposite in the other.

#### 2.6.2 Fractal dynamics of regional neural activity during MI

One of the additional benefits of utilizing rtDCCA to its full, multiscale extent that it also provides real-time estimates on the univariate DFA scaling exponents at every time instance (Kaposzta et al., 2022). Even though the scale-free (i.e., *fractal*) nature of neural dynamics is well-known, its neurophysiological relevance is less understood (He, 2014). Therefore, we explored how the DFA scaling exponent (characterizing long-range autocorrelation in the signal) changes over the cortex when performing left and right MI. For this analysis we evaluated broadband filtered data (4th order Butterworth filter with cutoff frequencies 1 and 45 Hz) at scales ranging from *s* = 16 to *s* = 256 in dyadic steps. The DFA scaling exponent was obtained via OLS regression of log-transformed fluctuation values on log-transformed scale (Peng et al., 1995; Hartmann et al., 2013). Note that in this analysis we utilized the traditional DFA formula outlined in Equations 4–6, including cumulative summation (Equation 4) as the first step (Peng et al., 1995; Hartmann et al., 2013). DFA exponents were contrasted on a location-by-location basis using paired *t*-test or Wilcoxon signed rank test, depending on the normality of the data as assessed via Lilliefors test.

## 3 Results

### 3.1 Offline dataset

Results regarding sample-wise Cohen's κ are summarized in Table 1, while those for accuracy are presented in Supplementary Table S1. Highest accuracy and Cohen's κ values among the three decoders are highlighted in bold for each evaluation pipeline. In general, the proposed DCCA-Riemannian-MDM pipeline yielded the highest grand average performance metrics in all three evaluation schemes when using detrending scales larger than *s* = 64 data points, while for *s* = 128 and above performance appeared to be plateauing. Rebiasing boosted the performance for all methods, while adaptive rebiasing—mimicking an online scenario—performed almost equally as well. Since detrending at scales *s* = 32 and *s* = 64 eventually hampered performance compared to Riemannian-MDM and Cov-CSP-LDA, we do not consider then any further, instead we report statistics for *s* = 128, with having found nearly identical results for *s* = 256 and *s* = 512 (see Supplementary material). Note that since the number of test examples was not necessarily balanced, Cohen's κ provides a more accurate characterization of classification performance. Therefore, we present the corresponding *p*-values in the main text while those regarding accuracy are reported in the Supplementary material. We have not found a significant main effect in the offline evaluation scheme, indicating no difference between the three decoders (noting that *post-hoc* pairwise comparisons indicated a tendency better performance of the DCCA-based decoder compared to the vanilla Riemannian approach with *p* = 0.0386, unadjusted). After rebiasing, however, Cov-CSP-LDA appeared to underperform the Riemannian geometry-based classifiers (main effect: *p* = 0.0124). Pairwise comparisons indicated that utilizing the DCCA matrix instead of the SCM provided a significant improvement (0.2190 ± 0.1825 vs. 0.2316 ± 0.1823, *p* = 0.0324, FDR-asjusted). No difference was found between Riemannian-MDM and Cov-CSP-LDA (*p* = 0.1841, FDR-adjusted), however DCCA-Riemannian-MDM expressed a tendency for outperforming the latter (*p* = 0.1061 following FDR-adjustment). Finally, in the pseudo-online case we again found a significant main effect (*p* = 0.0242). However, the improvement over the vanilla Riemannian approach by using DCCA was in the same range (0.2110 ± 0.1717 vs. 0.2206 ± 0.1670) and not statistically significant (*p* = 0.1701, adjusted). Also, the two Riemannian approaches exhibited marginally better performance than Cov-CSP-LDA following FDR-adjustment (*p* = 0.0837 and *p* = 0.0873 for DCCA-Riemannian-MDM and Riemannian-MDM, respectively). In summary, these results suggest that replacing the SCM with the DCCA matrix might introduce a small but stable improvement, which also outperformed the popular CSP-based method for MI detection.

**Table 1:** Offline performance.

**Cohen's** κ				
**Method**	**Scale**	**Offline**	**Offline rebias**	**Adaptive rebias**
Riemannian-MDM		0.1533 ± 0.1455	0.2190 ± 0.1825	0.2110 ± 0.1717
DCCA-Riemannian-MDM	*s* = 32	0.1088 ± 0.0950	0.1462 ± 0.1362	0.1406 ± 0.1301
	*s* = 64	0.1401 ± 0.1294	0.1889 ± 0.1619	0.1774 ± 0.1472
	*s* = 128	0.1647 ± 0.1546	0.2316 ± 0.1823	0.2193 ± 0.1671
	*s* = 256	0.1645 ± 0.1554	0.2314 ± 0.1830	**0.2206** ± **0.1670**
	*s* = 512	**0.1653** ± **0.1552**	**0.2318** ± **0.1826**	0.2205 ± 0.1670
Cov-CSP-LDA		0.1586 ± 0.1886	0.1853 ± 0.1929	0.1694 ± 0.1707

Scales for local detrending are given in data points. Bold indicates the highest performance setting in each column.

### 3.2 Online performance

According to the outcomes of the offline evaluation, performance did not improve any further by increasing the detrending scale beyond *s* = 128. Therefore, we decided to set *s* = 128 in the online evaluation pipeline as well, in order to maximally exploit the denoising effect of local detrending.

Sample-wise performance results (combining all five runs) for each participant are presented in Table 2. Overall, the DCCA-Riemannian-MDM decoder performed at 76.00 ± 8.07% on the group level. The lowest performance (Subject #5) was 65.41% sample-wise accuracy and 0.3092 Cohen's κ, while those of the best performing subject (#1) were 89.70% accuracy with 0.7942 Cohen's κ. Notably, all subjects could operate the BCI well above chance level (see Table 2), as well as Cohen's κ indicated fair, moderate and substantial decoding performance for two (#5 and #6), four (#3, #4, #7 and #8) and two (#1 and #2) participants, respectively. Bar dynamics data indicated that users were able to control the BCI and received consistent feedback, as they were able to drive the bar toward the correct direction for most of the time (in 82.99 ± 7.86% of active epochs).

**Table 2:** Sample-wise performance measures.

**Sample-wise performance**				
**Subject ID**	**Accuracy**	**Chance level**	**Bar dynamics**	**Cohen's** κ
1	89.70%	52.30%	94.08%	0.7942
2	84.31%	52.22%	91.26%	0.6854
3	74.67%	54.72%	83.92%	0.4945
4	76.92%	58.00%	87.44%	0.5363
5	65.41%	53.89%	73.68%	0.3092
6	67.09%	53.66%	71.97%	0.3414
7	73.87%	59.73%	79.69%	0.4803
8	76.02%	60.76%	81.87%	0.5190
AVG	76.00%	55.66%	82.99%	0.5200
STD	8.07%	3.36%	7.86%	0.1610
fMAX	89.70%	60.76%	94.08%	0.7942
MIN	65.41%	52.22%	71.97%	0.3092

AVG, average; STD, standard deviation; MAX, maximum value; MIN, minimum value.

Command delivery results are summarized in Table 3. All users were able to successfully deliver commands at a level surpassing chance by a large margin, with two participants even achieving perfect command delivery performance when excluding timeouts (i.e., 100% *Acc*_*comp*_). This also held for approximated accuracy, as with a total trial number of *n* = 100 and a balanced setting (50 − 50 for left and right) the global chance level was obtained as 65%. Normalized Cohen's κ values indicated at least moderate or substantial (two participants each) performance, while four users managed to produce κ_*norm*_ values surpassing 0.8. These results provide further support to the efficacy and robustness of the DCCA-Riemannian-MDM decoder.

**Table 3:** Command delivery performance measures.

**Command delivery performance**					
**Subject ID**	*Acc*_*approx*_	*Acc*_*comp*_	**Chance level**	**Timeouts**	**Cohen's** κ_*norm*_
1	99%	100%	65.96%	6	0.9400
2	97%	100%	66.67%	19	0.8100
3	94%	98.85%	66.67%	13	0.8498
4	89%	98.55%	68.12%	31	0.6700
5	82%	87.67%	68.49%	27	0.5507
6	82%	83.75%	67.50%	20	0.5377
7	90%	96.00%	68.00%	25	0.6889
8	95%	96.81%	65.96%	6	0.8797
AVG	91.00%	95.20%	67.17%	18.38	0.7409
STD	6.46%	6.11%	0.99%	9.38	0.1515
MAX	99%	100%	68.49%	31	0.9400
MIN	82%	83.75%	65.96%	6	0.5377

*Acc*_*approx*_, approximate accuracy; *Acc*_*comp*_, complete accuracy; Cohen's κ_*norm*_, normalized Cohen's κ value; AVG, average; STD, standard deviation; MAX, maximum value; MIN, minimum value.

### 3.3 Validation on independent MI data

Even though the offline evaluation implied an improvement in performance by our proposed approach over the vanilla Riemannian-MDM decoder, some of these results were only tendential and did not confirm the beneficial effect of using DCCA in a statistically robust manner. Therefore, it was imperative to confirm the validity of the DCCA-Riemannian-MDM method on a dataset that has sufficient sample size, as well as do not suffer from the possible confounding effects of between-session or feedback-introduced non-stationarities (see Section 4). Sample-wise Cohen's κ results obtained on the EEG Motor Movement/Imagery Dataset v1.0.0 for the left vs. right hand MI scenario are presented in Table 4, while those regarding both hands vs. both feet MI and 4-class MI are presented in Supplementary Tables S4, S5, along with sample-wise accuracy (Supplementary Table S3). These results show a pattern much similar to that presented in Table 1. In that, the performance of DCCA-Riemannian-MDM was inferior/comparable at scales *s* = 10 and 20 to the other classifiers, while for *s* = 40 and above, the DCCA-Riemannian-MDM outperformed both Riemannian-MDM and Cov-CSP-LDA in all three evaluation schemes. Precisely, for *s* = 40 in the Offline evaluation scheme, we found a significant main effect of decoder (*p* < 0.0001), and *post-hoc* pairwise comparisons indicated a better performance for DCCA-Riemannian-MDM (0.2461 ± 0.2293) when compared to both Riemannian-MDM (0.1979 ± 0.2079, *p* < 0.0001, FDR-adjusted) and Cov-CSP-LDA (0.2187 ± 0.2262, *p* = 0.0405, FDR-adjusted). In the Offline rebias scheme, along with a significant main effect (*p* < 0.0001), the DCCA-Riemannian-MDM decoder performed the best (0.2811 ± 0.2436 vs. 0.2207 ± 0.2297, *p* < 0.0001, FDR-adjusted against Riemannian-MDM and 0.2811 ± 0.2436 vs. 0.2414 ± 0.2390, *p* = 0.0012, FDR-adjusted against Cov-CSP-LDA). Finally, similar results were also obtained in the Adaptive Rebias scheme (main effect: *p* = 0.0001), with the DCCA-Riemannian-MDM decoder yielding significantly better performance (0.2630 ± 0.2320) when compared to Riemannian-MDM (0.2068 ± 0.2213, *p* < 0.0001, FDR-adjusted) and Cov-CSP-LDA (0.2237 ± 0.2327, *p* = 0.0002, FDR-adjusted). Results at scales *s* = 80 and *s* = 160 were comparable and are presented in the Supplementary material. Furthermore, results obtained in the both hands vs. both feet MI and 4-class MI scenarios expressed a similar pattern, with DCCA-Riemannian-MDM significantly outperforming the other two approaches under all three evaluation schemes (see Supplementary material).

**Table 4:** Offline performance on the EEG Motor Movement/Imagery Dataset v1.0.0 in case of left vs. right hand MI.

**Left vs. right hand MI**				
**Method**	**Scale**	**Offline**	**Offline rebias**	**Adaptive rebias**
Riemannian-MDM		0.1970 ± 0.2079	0.2207 ± 0.2297	0.2068 ± 0.2213
DCCA-Riemannian-MDM	*s* = 10	0.1639 ± 0.1529	0.1759 ± 0.1571	0.1523 ± 0.1431
	*s* = 20	0.2184 ± 0.1958	0.2410 ± 0.2050	0.2228 ± 0.1950
	*s* = 40	**0.2461** ± **0.2293**	0.2811 ± 0.2436	0.2630 ± 0.2320
	*s* = 80	0.2457 ± 0.2292	**0.2813** ± **0.2436**	0.2628 ± 0.2327
	*s* = 160	0.2456 ± 0.2290	**0.2813** ± **0.2434**	**0.2631** ± **0.2326**
Cov-CSP-LDA		0.2187 ± 0.2262	0.2414 ± 0.2390	0.2237 ± 0.2327

Scales for local detrending are given in data points. Bold indicates the highest performance setting in each column.

### 3.4 Detrended cross-correlation patterns during MI

**Figure 2:**
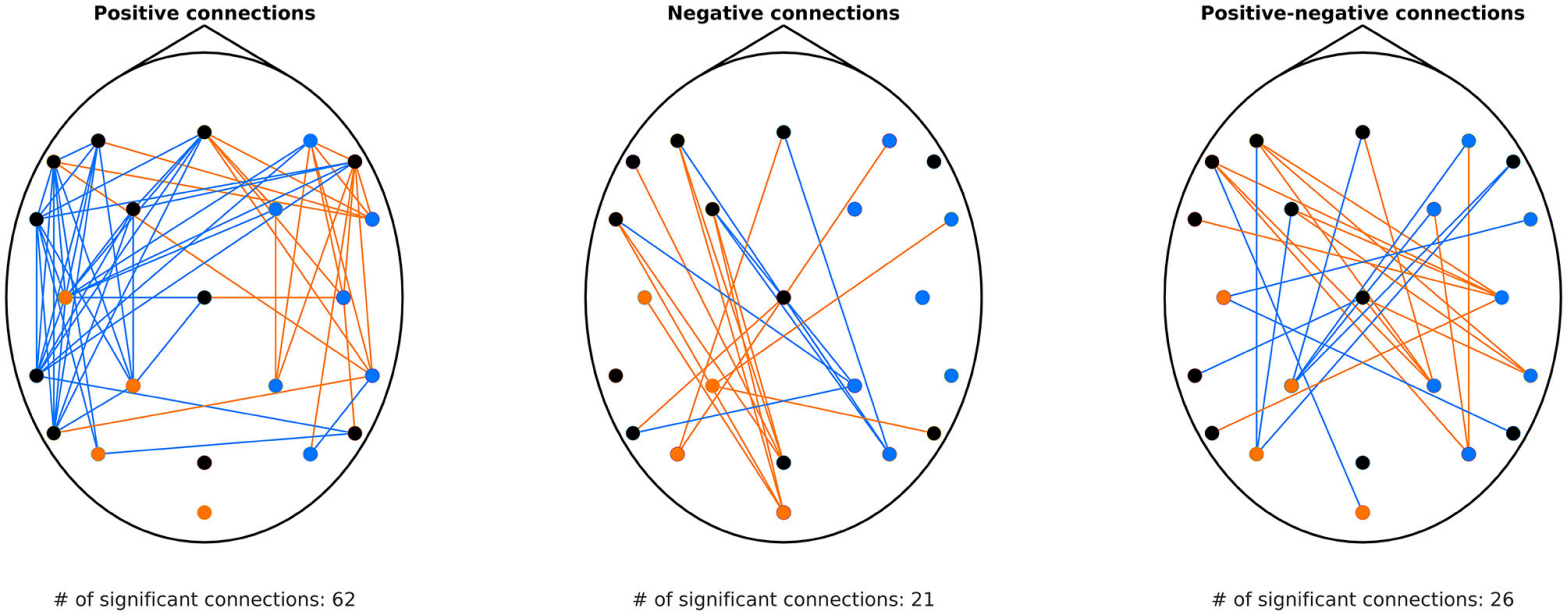
Connections with significantly different DCCC between left and right MI. The **(left)** panel shows connections characterized by positive DCCC under both conditions. Orange indicates *DCCC*_*left*_ > *DCCC*_*right*_, while blue the opposite. Similarly, the **(middle)** panel shows connections where DCCC was found negative in both left and right MI. Orange links denote connections where *DCCC*_*left*_ < *DCCC*_*right*_ (i.e., stronger anticorrelation), while blue links indicate the opposite case. The **(right)** panel shows connections that are characterized with DCCC values of opposite sign under the two conditions. Orange edges denote connections where DCCC was positive during left MI but negative during right MI, and blue edges vice versa. On all three panels, dots indicate the EEG channels, with orange color indicating higher detrended fluctuation in left compared to right MI, blue color indicating the opposite, and black color denoting no difference at the given cortical region. DCCC, detrended cross-correlation coefficient; MI, motor imagery; EEG, electroencephalography; *DCCC*_*left*_, DCCC value of the connection during left MI; *DCCC*_*right*_, DCCC value of the connection during right MI.

Results of this analysis are shown in Figure 2. All three panels indicate a strong lateralization: regional fluctuation (as indicated by the dot color) was found lower on the contralateral hemisphere (e.g., during left MI detrended fluctuation decreased over the right hemisphere). These changes were localized mostly over the sensorimotor regions (C3 and C4), also involving parietal electrodes (CP1, CP2, P3, P4). These results were expected and in line with the contralateral ERD established in the literature and confirmed by the BLP analysis (see Supplementary material). Regarding connectivity, it appears that during MI, even though variance in the contralateral motor cortex decreases (as seen at C4 and C3 for left and right MI, respectively), its activity becomes more positively correlated to that of frontal and temporal regions of the same hemisphere (Figure 2, left panel). This pattern is also observable in anticorrelated connections (Figure 2, middle panel), involving more parietofrontal connections, whose DCCC became less negative. Finally, the right panel of Figure 2 indicates that inter-hemispheric connections of motor regions contralateral to the MI task tend to switch from an anticorrelated to a positively correlated nature, also indicating that activity of the ipsilateral motor cortex is negatively correlated to contralateral brain regions. Additionally, as decoding was based on DCCA and not DCCC values, we performed the same analysis on DCCA matrices; the obtained results were in line with those of DCCC (see Supplementary Figure S3), implying that classification in essence was based on these emerging neural patterns.

### 3.5 Regional fractal dynamics

**Figure 3:**
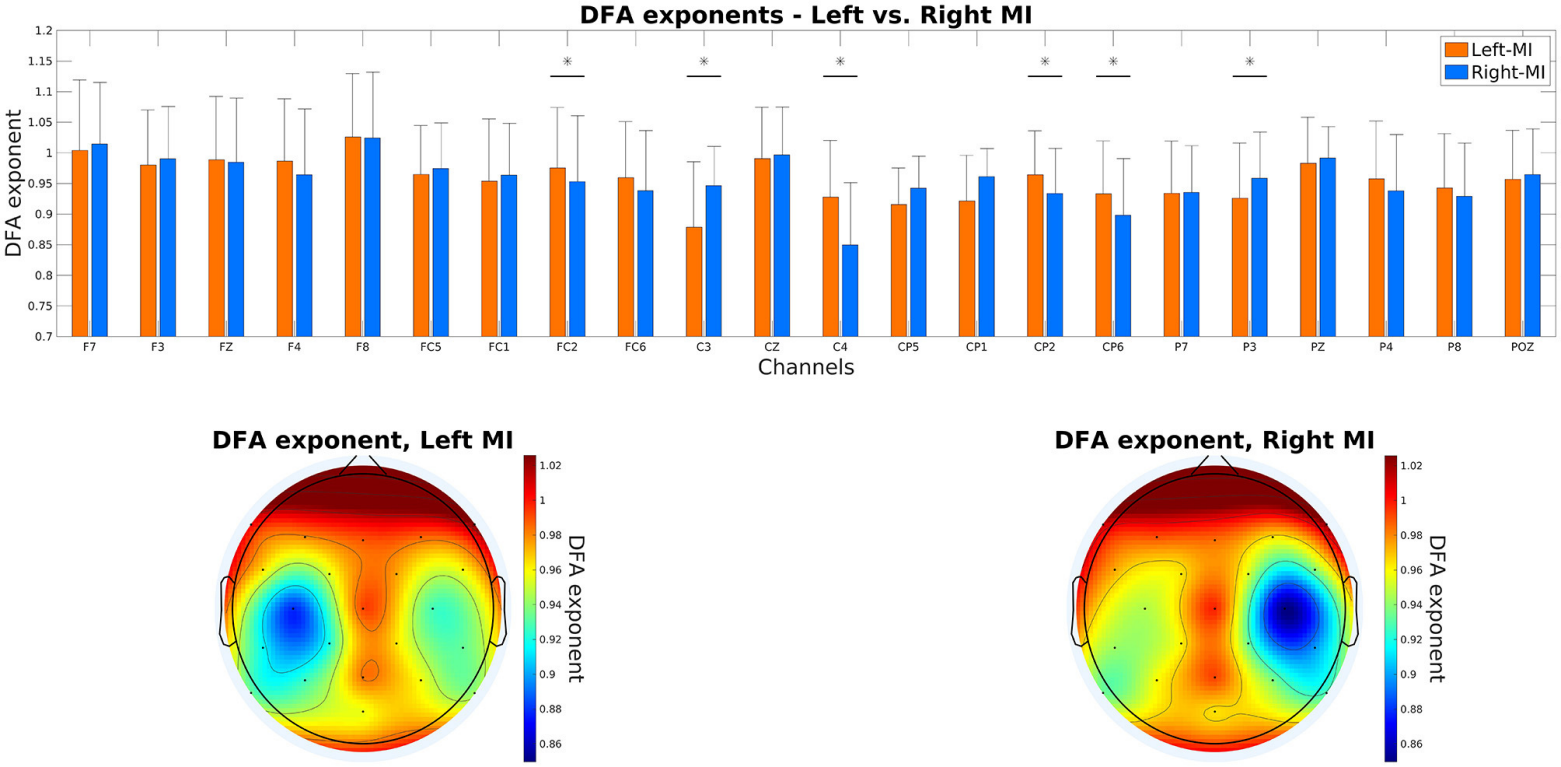
DFA scaling exponents over the cortex. The top panel shows the exponents of all cortical locations, with a vertical bar and asterisk symbol denoting significant difference between left and right MI. The bottom panels show the topoplots generated from the scaling exponents under left (left panel) and right (right panel) MI. DFA, detrended fluctuation analysis; MI, motor imagery.

Results are shown in Figure 3. DFA exponents were found significantly increased/decreased over the contralateral/ipsilateral motor cortex for the given MI task, i.e., during left MI, the scaling exponent was higher over C4 when compared to right MI (0.9278 ± 0.0926 vs. 0.8497 ± 0.1016, respectively, *p* < 0.0001), while the opposite was true for C3 (0.8785 ± 0.1070 vs. 0.9465 ± 0.0639 for left and right MI, respectively, *p* = 0.0348).

## 4 Discussion

In this study we proposed DCCA to replace the SCM in Riemannian geometry-based classification schemes. Results obtained from offline analysis of in-house EEG data implied that the introduction of DCCA might improve MI detection accuracy, which was then confirmed on an independent, publicly available MI dataset in a statistically robust manner. Furthermore, we demonstrated for the first time that DCCA can be computed online and thus utilized in a real-time BCI application, yielding a decoder with high performance.

### 4.1 The DCCA-Riemannian-MDM approach

When analyzing the offline in-house dataset, there was no significant main effect of decoder in the vanilla pipeline (i.e., without rebiasing and assessing the sole effect of DCCA). In general, performance of all three decoders were modest—with sample-wise accuracy not even surpassing 60% —, especially in contrast to what was observed during online testing. Applying rebiasing (Kumar et al., 2019, 2024) ameliorated this issue to some extent, boosting the performance of the Riemannian geometry-based pipelines above 60% and implying the DCCA-Riemannian-MDM decoder as superior. This effect stems from the structuring of the dataset analyzed offline in this study; namely, training and testing data was recorded in two independent sessions, on two consecutive days. It also highlights one of the main challenges of non-invasive BCIs: neural data collected on separate sessions are highly non-stationary, even when obtained from the same individual (Arvaneh et al., 2013), severely affecting stable long-term performance (Perdikis et al., 2018). In fact, one of the strengths of Riemannian geometry-based classification approaches lie in their allowance for domain matching across various recording sessions (Zanini et al., 2017; Rodrigues et al., 2018). Furthermore, this adaptation can be carried out online in a completely unsupervised manner, i.e., without knowing ground truth labels of the incoming test examples (Kumar et al., 2024). Combining rebiasing with DCCA, our proposed approach managed to achieve a between-session performance comparable to those obtained with sophisticated deep learning models evaluated on the same exact dataset (Liu et al., 2023).

Nevertheless, the offline analysis of our dataset with a relatively low sample size (*n* = 18) and including between-session non-stationarities only indicated a tendency, but not confirmation of performance improvement by introducing the DCCA matrix. Therefore, to ameliorate this shortcoming and to more robustly assess its utility, it was important to also evaluate the DCCA-Riemannian-MDM decoder on a large (*n* = 103), single-session dataset (Schalk et al., 2004). Outcomes of this analysis (Table 4, see also Supplementary material) clearly demonstrated that replacing the SCM with the DCCA matrix not only results in a significant improvement in performance in the Riemannian geometry-based framework, but it also outperforms the popular CSP-based decoding approach. Finally, we also re-evaluated the dataset collected for the online demonstration in the same manner as for the offline data. Results of this analysis (see Supplementary Table S2) reflected those of the former (presented in Table 1, with the addition that output measures reflected the better performance, as expected. Even though statistical evaluation did not identify significant differences between the methods after adjusting for multiple comparisons (see Supplementary material). This was, however, most likely a consequence of the low sample size (*n* = 8), as when observing performance measures on the individual level, Cohen's κ values were higher in the DCCA-Riemannian-MDM when compared to the Riemannian-MDM pipeline for eight out of eight participants. One remark must be made here: even though this dataset did not contain between-session non-stationarities, confounding effects could still be introduced by the adminstered online feedback, as it is established that BCI users adapt their mental strategy based on the feedback they receive (Biasiucci et al., 2018; Perdikis et al., 2018). Varying the scale parameter of DCCA revealed that it is a hyperparameter to be tuned, as it harmed classification accuracy by removing valuable information on smaller scales. Nevertheless, *s* = 128 (corresponding to 0.25 s) appears as a suitable choice for MI-based applications, as confirmed by the offline analysis of both datasets. Nevertheless, this might be an important aspect and plausible drawback of the proposed approach, that might limit its applicability on other BCI modalities utilizing e.g., event related potentials, where time domain information and waveform features are key.

Even though the number of studies proposing novel techniques for improving MI-BCI performance is yet increasing, most of the results are obtained via offline analysis and only a small proportion of these methods are tested in a true online environment [see e.g., Singh et al. (2021) for a recent review]. In addition with varying applied MI paradigms among works, therefore, it is difficult to objectively assess the online performance of our approach and to put it into perspective regarding the related literature. Furthermore, in this project we had two main goals: (*i*) to evaluate the plausible beneficial effect of utilizing DCCA instead of sample covariance matrix in the Riemannian framework, and (*ii*) to demonstrate that DCCA could be computed in real time using the formula of Kaposzta et al. (2022). Hence, for the online demonstration we recruited individuals who had prior experience with MI, most likely contributing to the high online performance observed, as well as we did not record a control group using a benchmark classification pipeline (i.e., vanilla Riemannian-MDM) online. Regardless, the DCCA-Riemannian-MDM decoder was operated online with promising performance, validating future research efforts to redeem the aforementioned limitations. In fact, it has been stressed previously that operating a BCI itself is a skill to be learned, with the ability of the user to control the application being just as important as the decoding algorithm providing online feedback (Perdikis et al., 2018; Perdikis and Millán, 2020; Silversmith et al., 2021; Tonin et al., 2022). Therefore, it appears as an interesting research endeavor to asses how the DCCA-Riemannian-MDM approach fosters online skill acquisition in comparison to other methods.

### 4.2 Neural patterns and DCCA during MI task performance

It is well established that MI evokes an event-related desynchronization (ERD) in the α (8–12 Hz) and β (13–30 Hz) frequency ranges that is most prominent over the contralateral sensorimotor regions (Pfurtscheller et al., 1997; Hwang et al., 2013). Therefore, as a proof of concept, we first confirmed this notion on our dataset via computing integrated band limited power (BLP) using Welch's method in the corresponding frequency ranges. Our results confirmed the expected neural patterns in both cases, supporting the participants' correct approach to the MI tasks (see Supplementary Figures S1, S2).

Regarding fractal connectivity and DCCA/DCCC, numerous previous studies explored how connectivity changes during MI performance [see e.g., Hamedi et al. (2016) for a review]. Drawing an exact comparison between our results and those reported previously is difficult, given that most studies utilized other connectivity estimators (e.g., phase synchronization or directed partial coherence), estimating connectivity based on fundamentally different principles. Nevertheless, several studies identified increased connectivity in the contralateral hemisphere during MI performance (Wang et al., 2006; Chung et al., 2011; Li et al., 2013). In contrast, Zhang et al. (2014) observed that within-hemisphere connectivity increases on the ipsilateral side relative to the MI task; however, we only found this pattern for anticorrelated, while the opposite for positive connections. Brunner et al. (2006) also stressed the importance of interhemispheric connections for MI classification, though in our analysis this pattern appeared most relevant for those connections whose nature (anticorrelated/correlated) was different during left and right MI. In summary, our analysis identified strong lateralization in connectivity patterns, supporting the notion of increased contralateral connectivity during MI. Furthermore, we revealed that correlated and anticorrelated patterns expressed markedly different topologies during left and right MI.

### 4.3 Fractal dynamics of neural activity during MI

Even though here we only compared the MI conditions to each other and not to baseline resting-state, our results are in line with those found by Wairagkar et al. (2021), reporting on the increase of the DFA exponent over the contralateral (active) region during upper limb MI. This pattern is again in line with the observed contralateral ERD; a decrease in the power of higher-frequency activity (and equivocally, a decrease in variance of small time scale fluctuations) will result in a scaling function with steeper slope/higher scaling exponent (Eke et al., 2000, 2002). Notably, on Figure 3 it might first appear that there is a decrease in the DFA exponent in the ipsilateral motor cortex in contrast to surrounding regions; however, we have to stress that since the primary goal of this study was not to assess fractal scaling during MI, our measurement protocol did not include a baseline resting condition. While Wairagkar et al. (2021) have identified a similar pattern regarding left vs. right MI, they also found that the DFA exponent increased during task performance compared to the resting-state baseline over both hemispheres, rendering an ipsilateral decrease unlikely in our study. Supporting this notion, previous studies also reported lower resting-state fractal scaling exponents over the somatomotor when compared to other cortical networks (He, 2011; Racz et al., 2019) that might also depend of the temporal scale/frequency range of analysis (Racz et al., 2021). Nevertheless, this issue must be resolved by future research where fractal scaling during MI is contrasted with resting-state baseline activity. Other previous studies also have attempted to characterize and classify MI data based on the fractal scaling exponent, although with limited success (Chen et al., 2013; Rodríguez-Bermúdez et al., 2015). Chen et al. (2013) identified globally increased DFA scaling exponent in right compared to left MI, not expressing the lateralization observed in our data. Interestingly, the increase of the DFA exponent in activated brain regions identified by our analysis and by that of Wairagkar et al. (2021)—also observed by Wairagkar et al. (2019) for executed movements—is in contrast with previous findings to some extent: it has been shown using multiple imaging techniques that the power-law scaling exponent of neural activity decreases over cortical regions activated by task performance (He et al., 2010; He, 2011). However, this difference was only statistically significant in frequencies below 10 Hz (Miller et al., 2009). These inconsistencies among reported findings regarding fractal scaling might contribute to poor classification performance of previous attempts. Furthermore, fractal analysis of EEG signals is computationally expensive in most cases (Racz et al., 2020; Czoch et al., 2023), diminishing its appeal for online BCI applications. On the other hand, univariate real-time DFA (Hartmann et al., 2013) and especially rtDCCA (Kaposzta et al., 2022) provide efficient ways for obtaining the DFA scaling exponents of multiple channels simultaneously in real time, allowing for future testing of decoders utilizing them as features.

As the exact origin and neurophysiological relevance of scale-free brain activity is not yet fully understood, resolving contradictions regarding our findings and those of previous studies requires further research beyond the scope of this current paper. In general, a higher DFA scaling exponent implies stronger long-term autocorrelation in neural activity, as well as values above 0.5 indicate a persistent process, i.e., amplitude changes in one direction in the past are likely to be followed by future changes in the same direction (Eke et al., 2002). Scale-free activity in biological systems (also often referred to as the 1/*f* characteristic) are hypothesized to originate from stochastic feedback loops of antagonistic effect (Ivanov et al., 1998). With respect to 1/*f* neural dynamics it might reflect the superposition of activity generated by neuronal populations of varying sizes, inversely proportional to the characteristic frequency of their activities (Buzsaki and Draguhn, 2004). With this in mind, an increase in the DFA exponent in the contralateral hemisphere might reflect the involvement of larger neuronal assemblies, in line with the observed increase in large-scale connectivity (see Figure 2, left panel). The changing balance in incoming inhibitory and excitatory activity might also be reflected by how connections tend to turn from anticorrelated to correlated on the cortical site opposite to the MI task (see Figure 2, right panel). However, it must be noted that DCCA (DCCC) analysis is unable to detect directionality of the relationship, as well as the nature of anticorrelated neural activity between neuronal populatioins is difficult to interpret (Bullmore and Sporns, 2009). In any case, our results provide further support for the functional significance of fractal brain activity, that also appears to be significant in MI task performance.

### 4.4 Limitations and future perspectives

The two main goals of this study were to evaluate if DCCA can improve Riemannian geometry-based classification, and to test the DCCA-Riemannian-MDM decoder online. The study was designed accordingly, while keeping complexity of the pipeline to the minimum in order to avoid confounding factors. In consequence, our intent was not to propose a decoder with state-of-the-art best performance. Detection accuracy could likely be further improved with the introduction of additional processing steps, such as Fisher Geodesic Filtering (Barachant et al., 2010b), or utilizing classification schemes more elaborate than simple MDM, such as RBNNet (Liu et al., 2023). It is widely recognized that the ability to control a BCI in the BCI-näive population varies greatly, with often studies retrospectively sorting their cohort as “good-” and “bad-performing” participants (Ahn and Jun, 2015). For the online demonstration we recruited individuals with prior experience in operating an MI-BCI. Although this introduces a positive bias in the online performance, we wanted to avoid the confounding effect of recruiting users unable to control the application immediately. Similarly, we also have not considered an independent control group utilizing a benchmark Riemannian decoder for online testing. Comparing online performances on an absolute scale can be challenging, and a common difficulty in BCI research. Individual variability in baseline BCI performance varies greatly among users, with some being unable to gain BCI control even after practicing (Reichert et al., 2015). Therefore, a group-level difference in online performance might simply reflect a different proportion of 'slow BCI learners' in one group, a notion that is very hard to control for without arbitrarily influencing the outcomes. A possible solution would be to have the same individuals operate both decoders in an alternating manner; however, this would raise the issues of time dependence of performance [i.e., learning effect (Perdikis et al., 2018; Tonin et al., 2022)], as well as inconsistent feedback from different decoders could confuse the user and thus hamper BCI control (Biasiucci et al., 2018). Even though we found strong statistical support for the superiority of the DCCA-Riemannian-MDM approach over vanilla Riemannian-MDM and Cov-CSP-LDA when evaluated on the EEG Motor Movement/Imagery Database (Goldberger et al., 2000), offline analysis of our in-house MI data mostly revealed tendential improvement in performance once adjusted for multiple comparisons. One of the possible reasons behind this could be the relatively low sample size in our sample. It should be noted, however, that all data (both offline and online) was recorded in a laboratory environment with users sitting idly in a comfortable chair, and therefore the level of artifacts can be assumed to be low. Most likely, the denoising effect of local detrending would be more prominent in environments better resembling real-world scenarios, where data can be contaminated by various sources (such as e.g., head movements, sweat, unconstrained eye movements); testing this is indeed one of our priorities in the future. It has also been stressed that BCI skill acquisition is a lengthy process that fundamentally depends on the consistency of the feedback provided on brain activity (Perdikis et al., 2018). In that regard, it would be interesting to see if the introduction of the DCCA matrix could foster faster subject learning by providing more consistent feedback.

Even though results from the *post-hoc* evaluation of the online-collected data convincingly indicated the involvement of relevant cortical locations (namely, C3 and C4), our analyses on fractal connectivity and regional activity were largely exploratory and therefore our interpretations should be treated with caution. We only had a small sample size of eight participants, as well as the study design and strategy was focused on online BCI applicability instead of drawing conclusions on undergoing neural processes. With this in mind, our results hopefully facilitate future research aiming on better understanding how functional connectivity and regional fractal dynamics alter during MI task performance, as well as how these two aspects of brain function might be related. Finally, we only explored the utility of the proposed DCCA-Riemannian-MDM approach for MI; it would be also important to assess its performance in other BCI use cases, such as detecting error related potentials (Barachant, 2014) or movement-related cortical potentials (Racz et al., 2023), where Riemannian geometry-based classification is frequently utilized. Namely, detection of event related potentials is often based on detecting a waveform in the time domain, which process could be also hampered by local detrending by removing parts of the target signal.

## 5 Conclusions

In this study we proposed DCCA to replace the sample covariance matrix in Riemannian geometry-based classification pipelines, using MI as an exemplary case. Offline analysis indicated consistent improvement in performance compared to vanilla Riemannian-MDM approach, as well as outperforming standard CSP-based classifier. Importantly, we successfully tested the proposed decoder online involving eight participants, achieving high performance. *Post-hoc* analysis of EEG data revealed distinctive connectivity patterns under left and right MI conditions, while fractal scaling of neural activity was also shown to alter over the contralateral motor regions (C3 and C4) during task performance. In conclusion, we propose the DCCA-Riemannian-MDM decoder as a suitable, high performing tool for future MI-based BCI studies.

## Data availability statement

The offline in-house dataset can be accessed at https://zenodo.org/records/10694. The online EEG datasets analyzed in this study are available at the corresponding author upon reasonable request. The EEG Motor Movement/Imagery Dataset v1.0.0 can be accessed via Physionet (Goldberger et al., 2000) at https://physionet.org/content/eegmmidb/1.0.0/. Matlab and Python implementations of the rtDCCA method are available at https://github.com/samuelracz/rsDCCA.

## Ethics statement

The studies involving humans were approved by Institutional Review Board, The University of Texas at Austin. The studies were conducted in accordance with the local legislation and institutional requirements. The participants provided their written informed consent to participate in this study.

## Author contributions

FR: Conceptualization, Data curation, Formal analysis, Investigation, Methodology, Project administration, Validation, Visualization, Writing—original draft, Writing—review & editing. SK: Conceptualization, Data curation, Formal analysis, Investigation, Methodology, Software, Writing—review & editing. ZK: Methodology, Software, Writing—review & editing. HA: Investigation, Visualization, Writing—review & editing. DL: Data curation, Writing—review & editing. RL: Data curation, Formal analysis, Methodology, Software, Writing—review & editing. AC: Software, Writing—review & editing. PM: Investigation, Methodology, Writing—review & editing. JM: Funding acquisition, Project administration, Resources, Supervision, Writing—review & editing.
