# Supplementary material for: Combining detrended cross-correlation analysis with Riemannian geometry-based classification for improved brain-computer interface performance
Source: Front Neurosci. 2024 Mar 14;18:1271831. doi: 10.3389/fnins.2024.1271831 (PMC10973000; doi:10.3389/fnins.2024.1271831)
Supplement: Supplementary file 1 [file Data_Sheet_1.PDF]

Supplementary Material - Combining detrended cross-correlation analysis with Riemannian geometry-based detection for improved brain-computer interface performance

1 OFFLINE RESULTS

1.1 Accuracy at s=128

Results are summarized in **Table S1**, while in the following we only detail significant differences. In the offline case no significant main effect was found, even though DCCA-Riemannian-MDM outperformed Riemannian-MDM ($p = 0.0386$, unadjusted). The main effect was significant in the Offline Rebias case ($p = 0.0124$), with DCCA-Riemannian-MDM outperforming Riemannian-MDM ($p = 0.0288$, FDR-adjusted). The main effect was also significant in the Adaptive Rebias scenario, with Cov-CSP-LDA being marginally outperformed by DCCA-Riemannian-MDM ($p = 0.0837$, FDR-adjusted).

1.2 s=256

Main effect was not significant for either Cohen's κ or Accuracy in the offline case. There was a significant main effect, however, in the Offline Rebias scenario for both metrics ($p = 0.0242$ in both cases), where DCCA-Riemannian-MDM outperformed Riemannian-MDM in terms of Accuracy ($p = 0.0369$, FDR-adjusted) and κ ($p = 0.0417$, FDR-adjusted). Main effect was also significant for the Adaptive Rebias pipeline ($p = 0.0173$ and $p = 0.0111$ for Accuracy and Cohen's κ, respectively); DCCA-Riemannian-MDM could be characterized with marginally higher Accuracy ($p = 0.0933$, FDR-adjusted) and κ ($p = 0.0747$, FDR-adjusted) than Cov-CSP-LDA.

1.3 s=512

No main effect was found in the Offline case. Main effect was significant during Offline Rebias ($p = 0.0242$ for both measures) where DCCA-Riemannian-MDM outperformed Riemannian-MDM ($p = 0.0252$, FDR-adjusted, for both measures). The main effect was significant for both Accuracy ($p = 0.0173$) and κ ($p = 0.0111$) in the Adaptive Rebias scheme; DCCA-Riemannian-MDM marginally outperformed Cov-CSP-LDA in terms of Accuracy ($p = 0.0837$, FDR-adjusted) and Cohen's κ ($p = 0.0747$, FDR-adjusted).

2 INDEPENDENT MOTOR IMAGERY DATASET

Accuracy results are reported in **Supplementary Table S3**. Results are well aligned with those reported for Cohen's κ in the main text. Notably, Performance values are comparable with those obtained from our in-house 2-class MI dataset (see e.g., **Supplementary Table S1**.At $s = 10$ (main effect: $p < 0.05$ for all evaluation schemes), DCCA-Riemannian-MDM performed significantly worse than the other two decoders ($p < 0.05$ in all cases, FDR-adjusted). At $s = 20$ we found a tendency for DCCA-Riemannian-MDM to outperform vanilla Riemannian-MDM (but not Cov-CSP-LDA), however these differences were found only marginally significant in most cases ($p < 0.1$, FDR-adjusted). When increasing the detrending scale

to $s = 40$ and above, the performance of DCCA-Riemannian-MDM became significantly better than both Riemannian-MDM and Cov-CSP-LDA. A significant main effect ($p < 0.0001$) was found in all three evaluation schemes, while post hoc pairwise comparisons confirmed the superior performance of DCCA-Riemannian-MDM ($p < 0.05$ in all cases, FDR-adjusted). Results obtained at $s = 80$ and $s = 160$ were equivalent in nature to those at $s = 40$. Notably, Cov-CSP-LDA outperformed Riemannian-MDM in the offline ($p = 0.345$, FDR-adjusted) and offline rebias ($p = 0.0495$, FDR-adjusted), but not in the adaptive rebias ($p = 0.0882$, FDR-adjusted) schemes.

2.1 Both hands vs. both feet MI

Results of this analysis is presented in **Supplementary Table S4**. In accordance with our results presented in the main text, DCCA-Riemannian-MDM underperformed the other classifiers at $s = 10$. There was a significant main effect of decoder in all evaluation schemes ($p < 0.0001$ in all cases). Post hoc analysis revealed, that DCCA-Riemannian-MDM underperformed underperformed the other two decoders in both Accuracy and Cohen's κ under all three evaluation schemes ($p < 0.0001$ in all cases, FDR-adjusted). Notably, Cov-CSP-LDA also performed better than Riemannian-MDM ($p = 0.0106$ and $p = 0.0178$ for Accuracy and κ, respectively, FDR-adjusted), however only in the basic Offline setting, while no statistical difference was identified between the two approaches in the Offline Rebias and Adaptive rebias pipelines ($p > 0.15$ in all cases, FDR-adjusted), implying similar performance. In case of $s = 20$, a main effect was only identified for Accuracy in the Offline setting ($p = 0.0255$) due to the slightly better performance of Cov-CSP-LDA when compared to Riemannian-MDM ($p = 0.0318$, FDR-adjusted), indicating comparable performance for the three decoders. At $s = 40$ we found a significant main effect of decoder for both Accuracy and Cohen's κ in all three evaluation schemes ($p < 0.0001$ in all cases). In the offline scheme, DCCA-Riemannian-MDM outperformed both Riemannian-MDM and Cov-CSP-LDA in terms of Accuracy and κ ($p < 0.0001$, in all cases, FDR-adjusted). Notably, Cov-CSP-LDA also performed better than Riemannian-MDM ($p = 0.0106$ and $p = 0.0178$ for Accuracy and κ, respectively, FDR-adjusted). A similar trend was observed in the Offline Rebias and Adaptive Rebias pipelines: DCCA-Riemannian-MDM performed significantly better than the other two decoders ($p < 0.0001$ in all cases, for both Accuracy and κ, FDR-adjusted), however, a significant difference was no longer found between Riemannian-MDM and Cov-CSP-LDA ($p > 0.1$ in all cases), indicating a comparable performance. Results obtained at $s = 80$ and $s = 160$ were identical (regarding significant differences) to those at $s = 40$, indicating the superior performance of DCCA-Riemannian-MDM compared to vanilla Riemannian-MDM and Cov-CSP-LDA. In summary, these results confirm those found for left vs. right hand MI, extending them with the notion that the DCCA-Riemannian-MDM approach generalizes well for two-class MI scenarios other than left vs. right MI.

2.2 4-class MI

Finally, we tested if the DCCA-Riemannian-MDM approach would perform well in not only two-, but in to multi-class MI problems too. For that purpose, in these evaluation schemes we included data from all four MI classes (left hand, right hand, both hands, both feet) and compared their performances under the three schemes as previously. Note that even though it can be implemented, the CSP method is not ideal for more than two class problems (Barachant et al., 2010), limiting the interpretability of results reported for the Cov-CSP-LDA decoder.

We found a similar pattern in the results (**Supplementary Table S5** as in the previous cases. In that, at $s = 10$ the DCCA-Riemannian-MDM decoder performed worse (main effect: $p < 0.05$ in all cases) than the other decoders ($p < 0.05$ in all cases for both Accuracy and κ, FDR-adjusted). No significant

main effect could be identified at $s = 20$, indicating similar decoder performance. For scales $s = 40$ (and greater), however, the DCCA-Riemannian-MDM decoder performed significantly better than the other two, as indicated by significant main effect and post hoc pairwise comparisons ($p < 0.0001$ in all cases, FDR-adjusted). Results at scales $s = 80$ and $s = 160$ were identical in nature. Notably, no significant difference was found between vanilla Riemannian-MDM and Cov-CSP-LDA ($p > 0.2$ in all cases, FDR-adjusted).

In conclusion, the DCCA-Riemannian-MDM demonstrated a robust improvement over the vanilla Riemannian-MDM decoder, as well as outperformed the considered gold standard CSP-based classification too. Furthermore, it generalized well to a 4-class MI scenario with reasonable performance.

REFERENCES

Barachant, A., Bonnet, S., Congedo, M., and Jutten, C. (2010). Common spatial pattern revisited by Riemannian geometry. In *2010 IEEE International Workshop on Multimedia Signal Processing*. 472–476

3 SUPPLEMENTARY TABLES AND FIGURES

Accuracy				
Method	Scale	Offline	Offline Rebias	Adaptive Rebias
Riemannian-MDM		$57.16 \pm 7.98\%$	$61.01 \pm 9.13\%$	$60.47 \pm 8.59\%$
DCCA-Riemannian-MDM	$s = 32$	$55.16 \pm 5.01\%$	$57.36 \pm 6.81\%$	$56.98 \pm 6.43\%$
	$s = 64$	$56.60 \pm 6.90\%$	$59.49 \pm 8.11\%$	$58.77 \pm 7.33\%$
	$s = 128$	$57.77 \pm 8.34\%$	$61.63 \pm 9.11\%$	$60.84 \pm 8.36\%$
	$s = 256$	$57.79 \pm 8.39\%$	$61.62 \pm 9.14\%$	**60.90 ± 8.36%**
	$s = 512$	$57.78 \pm 8.38\%$	**61.65 ± 9.12%**	$60.89 \pm 8.37\%$
Cov-CSP-LDA		**57.88 ± 9.94%**	$59.49 \pm 9.64\%$	$58.54 \pm 8.53\%$

Table S1. Offline performance - Accuracy. Scales for local detrending are given in data points.

Accuracy				
Method	Scale	Offline	Offline Rebias	Adaptive Rebias
Riemannian-MDM		$74.02 \pm 14.28\%$	$77.72 \pm 13.11\%$	$75.90 \pm 13.08\%$
DCCA-Riemannian-MDM	$s = 32$	$67.94 \pm 12.97\%$	$69.42 \pm 12.65\%$	$68.27 \pm 12.19\%$
	$s = 64$	$72.72 \pm 13.08\%$	$74.76 \pm 12.55\%$	$73.08 \pm 12.18\%$
	$s = 128$	$75.66 \pm 14.13\%$	$78.76 \pm 12.66\%$	**76.91 ± 12.58%**
	$s = 256$	$75.70 \pm 14.05\%$	**78.79 ± 12.66%**	$76.89 \pm 12.49\%$
	$s = 512$	$75.71 \pm 14.03\%$	$78.75 \pm 12.68\%$	**76.91 ± 12.48%**
Cov-CSP-LDA		**76.47 ± 14.03%**	$78.15 \pm 11.87\%$	$76.33 \pm 12.10\%$
**Cohen's κ**				
Method	Scale	Offline	Offline Rebias	Adaptive Rebias
Riemannian-MDM		$.4686 \pm .2933$	$.5523 \pm .2626$	$.5208 \pm .2588$
DCCA-Riemannian-MDM	$s = 32$	$.3520 \pm .2628$	$.3885 \pm .2536$	$.3699 \pm .2429$
	$s = 64$	$.4473 \pm .2644$	$.4944 \pm .2516$	$.4662 \pm .2419$
	$s = 128$	$.5031 \pm .2887$	$.5733 \pm .2534$	$.5412 \pm .2491$
	$s = 256$	$.5039 \pm .2871$	**.5739 ± .2534**	$.5408 \pm .2475$
	$s = 512$	$.5041 \pm .2867$	$.5732 \pm .2538$	**.5413 ± .2472**
Cov-CSP-LDA		**.5315 ± .2740**	$.5613 \pm .2375$	$.5295 \pm .2403$

Table S2. Re-evaluating the online dataset with the approaches utilized on the offline dataset. Scales for local detrending are given in data points.

Accuracy				
Method	Scale	Offline	Offline Rebias	Adaptive Rebias
Riemannian-MDM		$59.89 \pm 10.44\%$	$61.03 \pm 11.50\%$	$60.32 \pm 11.09\%$
DCCA-Riemannian-MDM	$s = 10$	$58.30 \pm 7.62\%$	$58.80 \pm 7.85\%$	$57.61 \pm 7.17\%$
	$s = 20$	$61.05 \pm 9.72\%$	$62.06 \pm 10.25\%$	$61.12 \pm 9.78\%$
	$s = 40$	**62.48 ± 11.35%**	$64.07 \pm 12.17\%$	$63.13 \pm 11.62\%$
	$s = 80$	$62.46 \pm 11.34\%$	**64.08 ± 12.17%**	$63.12 \pm 11.66\%$
	$s = 160$	$62.46 \pm 11.34\%$	**64.08 ± 12.17%**	**63.14 ± 11.66%**
Cov-CSP-LDA		$60.99 \pm 11.31\%$	$62.08 \pm 11.94\%$	$61.17 \pm 11.63\%$

Table S3. Accuracy for left vs. right hand MI on the EEG Motor Movement/Imagery Database v1.0.0. Scales for local detrending are given in data points.

Both Hands vs. Both Feet Motor Imagery				
**Accuracy**				
Method	Scale	Offline	Offline Rebias	Adaptive Rebias
Riemannian-MDM		$63.71 \pm 12.07\%$	$65.04 \pm 12.34\%$	$64.63 \pm 11.99\%$
DCCA-Riemannian-MDM	$s = 10$	$60.03 \pm 9.66\%$	$60.45 \pm 10.00\%$	$59.93 \pm 9.50\%$
	$s = 20$	$64.16 \pm 11.61\%$	$65.00 \pm 11.82\%$	$64.63 \pm 11.66\%$
	$s = 40$	**67.14 ± 13.52%**	$68.48 \pm 13.70\%$	**68.02 ± 13.34%**
	$s = 80$	**67.14 ± 13.54%**	$68.48 \pm 13.70\%$	$68.01 \pm 13.30\%$
	$s = 160$	$67.13 \pm 13.56\%$	**68.50 ± 13.70%**	$68.01 \pm 13.29\%$
Cov-CSP-LDA		$65.11 \pm 13.52\%$	$65.84 \pm 13.46\%$	$65.39 \pm 13.16\%$
**Cohen's κ**				
Method	Scale	Offline	Offline Rebias	Adaptive Rebias
Riemannian-MDM		$.2745 \pm .2393$	$.3003 \pm .2470$	$.2904 \pm .2405$
DCCA-Riemannian-MDM	$s = 10$	$.2010 \pm .1924$	$.2087 \pm .2000$	$.1981 \pm .1897$
	$s = 20$	$.2832 \pm .2316$	$.2994 \pm .2366$	$.2916 \pm .2330$
	$s = 40$	**.3427 ± .2693**	**.3691 ± .2741**	**.3591 ± .2663**
	$s = 80$	**.3427 ± .2697**	**.3691 ± .2742**	$.3589 \pm .2657$
	$s = 160$	$.3425 \pm .2700$	$.3695 \pm .2742$	$.3588 \pm .2655$
Cov-CSP-LDA		$.3008 \pm .2702$	$.3164 \pm .2694$	$.3065 \pm .2635$

Table S4. Offline performance obtained from both hands vs. both feet motor imagery data. Scales for local detrending are given in data points.

**4-class Motor Imagery**				
**Accuracy**				
Method	Scale	Offline	Offline Rebias	Adaptive Rebias
Riemannian-MDM		$34.55 \pm 10.81\%$	$35.46 \pm 12.73\%$	$34.20 \pm 10.65\%$
DCCA-Riemannian-MDM	$s = 10$	$31.64 \pm 8.79\%$	$32.41 \pm 10.64\%$	$31.14 \pm 7.48\%$
	$s = 20$	$34.90 \pm 10.46\%$	$36.03 \pm 12.69\%$	$34.58 \pm 10.35\%$
	$s = 40$	$37.19 \pm 12.15\%$	**$38.80 \pm 14.85\%$**	$37.05 \pm 12.41\%$
	$s = 80$	**$37.20 \pm 12.15\%$**	**$38.80 \pm 14.86\%$**	**$37.08 \pm 12.41\%$**
	$s = 160$	$37.18 \pm 12.16\%$	**$38.80 \pm 14.86\%$**	$37.07 \pm 12.42\%$
Cov-CSP-LDA		$34.29 \pm 11.88\%$	$35.08 \pm 13.41\%$	$34.32 \pm 11.70\%$
**Cohen's κ**				
Method	Scale	Offline	Offline Rebias	Adaptive Rebias
Riemannian-MDM		$.1269 \pm .1445$	$.1394 \pm .1698$	$.1227 \pm .1420$
DCCA-Riemannian-MDM	$s = 10$	$.0881 \pm .1158$	$.0986 \pm .1417$	$.0891 \pm .0995$
	$s = 20$	$.1312 \pm .1396$	$.1470 \pm .1689$	$.1279 \pm .1377$
	$s = 40$	$.1614 \pm .1623$	**$.1841 \pm .1977$**	$.1611 \pm .1650$
	$s = 80$	**$.1615 \pm .1624$**	$.1840 \pm .1977$	**$.1613 \pm .1651$**
	$s = 160$	$.1613 \pm .1625$	$.1840 \pm .1977$	**$.1613 \pm .1652$**
Cov-CSP-LDA		$.1239 \pm .1580$	$.1345 \pm .1790$	$.1245 \pm .1561$

Table S5. Offline performance obtained from both hands vs. both feet motor imagery data. Scales for local detrending are given in data points.

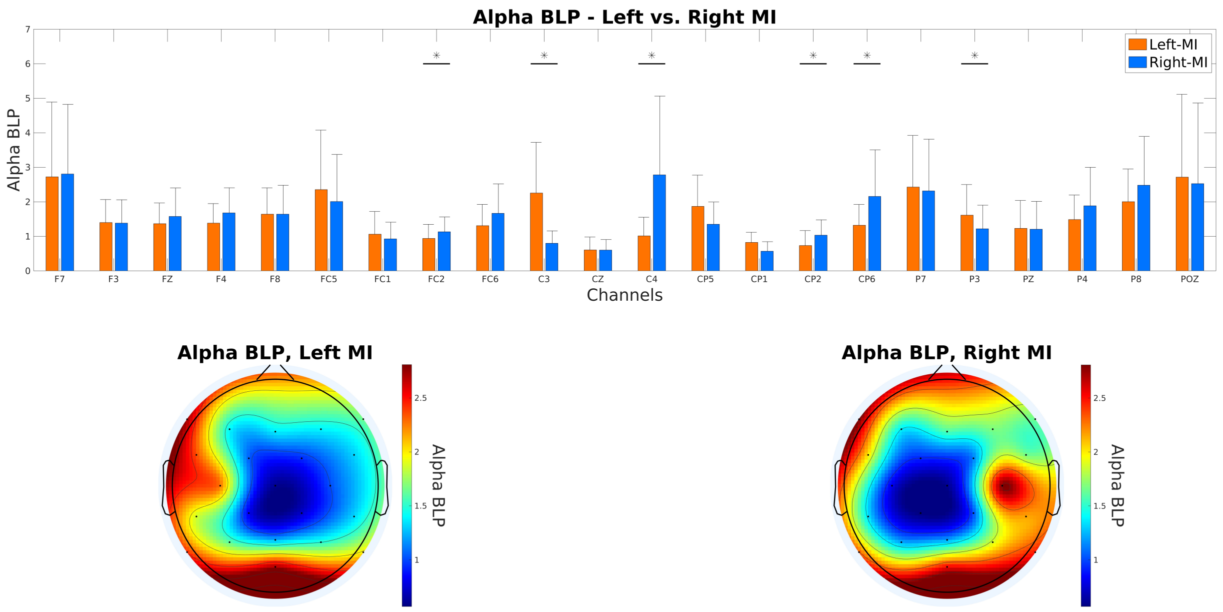

Figure S1. Alpha band-limited power over the cortex during left and right MI. The top panel shows the region-to-region pairwise comparisons with vertical bar and asterisk symbols indicating significant difference in alpha BLP between left and right MI. Lower panels show the topological distribution of alpha BLP during left (left panel) and right (right panel) MI. Note that this analysis was carried out on data re-referenced to the common average electrode, in order to better visualize the spatial distribution of BLP in the alpha $(8 - 12Hz)$ range. BLP: band-limited power; MI: motor imagery.

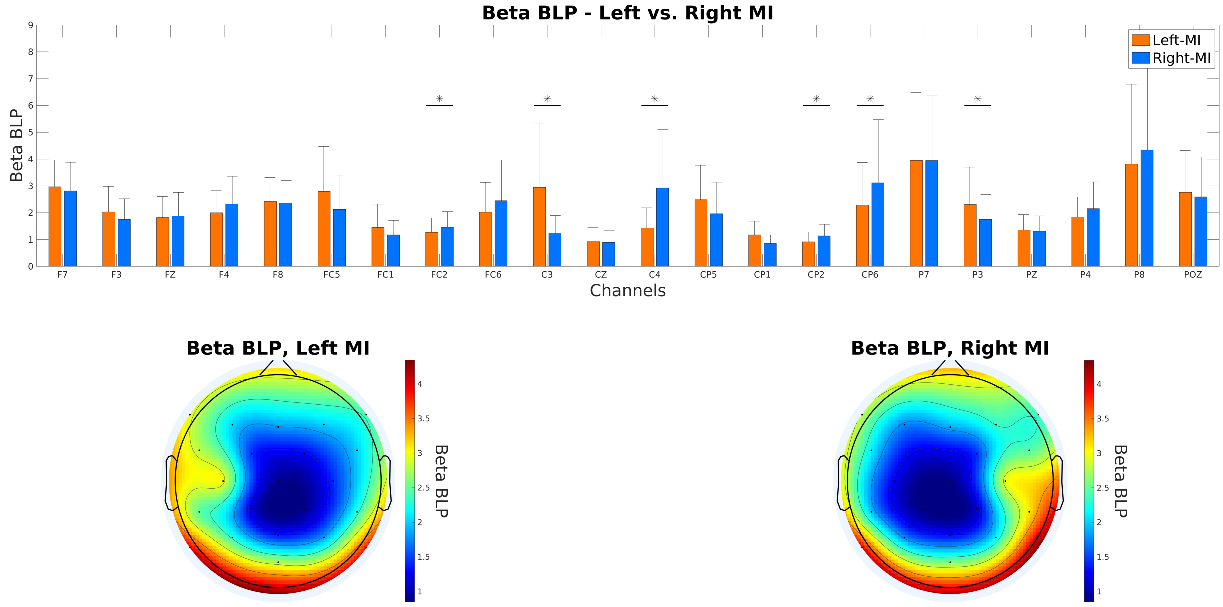

Figure S2. Beta band-limited power over the cortex during left and right MI. The top panel shows the region-to-region pairwise comparisons with vertical bar and asterisk symbols indicating significant difference in beta BLP between left and right MI. Lower panels show the topological distribution of beta BLP during left (left panel) and right (right panel) MI. Note that this analysis was carried out on data re-referenced to the common average electrode, in order to better visualize the spatial distribution of BLP in the beta $(13 - 30Hz)$ range. BLP: band-limited power; MI: motor imagery.

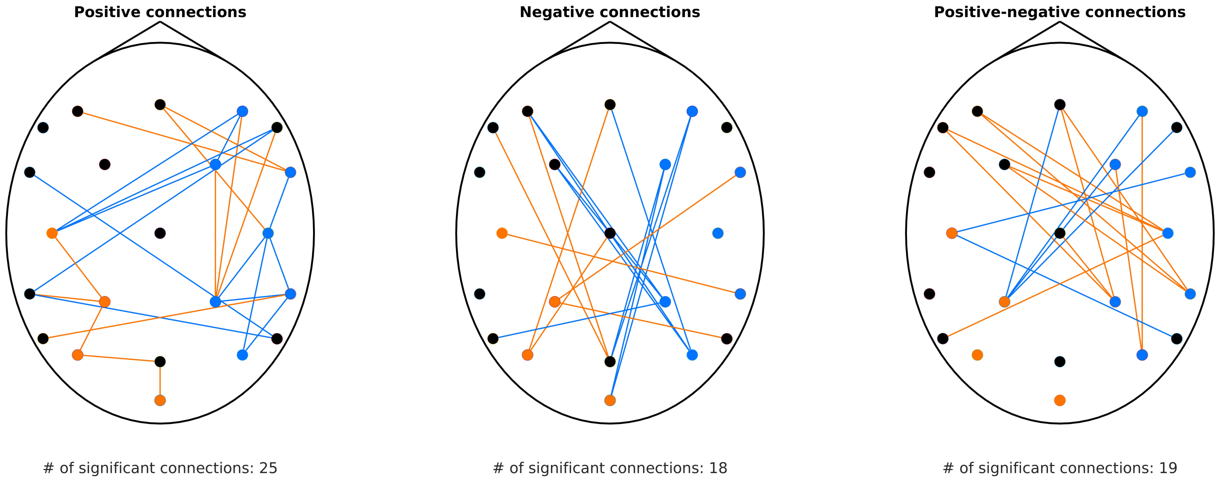

Figure S3. Connections with significantly different DCCA between left and right MI. The left panel shows connections characterized by positive DCCA under both conditions. Orange indicates $DCCA_{left} > DCCA_{right}$, while blue the opposite. Similarly, the middle panel shows connections where DCCA was found negative in both left and right MI. Orange links denote connections where $DCCA_{left} < DCCA_{right}$, while blue links indicate the opposite case. The right panel shows connections that are characterized with DCCA values of opposite sign under the two conditions. Orange edges denote connections where DCCA was positive during left MI but negative during right MI, and blue edges vice versa. On all three panels, dots indicate the EEG channels, with orange color indicating higher detrended fluctuation in left compared to right MI, blue color indicating the opposite, and black color denoting no difference at the given cortical region. DCCA: detrended cross-correlation analysis; MI: motor imagery; EEG: electroencephalography; $DCCA_{left}$: DCCA value of the connection during left MI; $DCCA_{right}$: DCCA value of the connection during right MI.
